# Anti-IL-5 and IL-5Ra: Efficacy and Safety of New Therapeutic Strategies in Severe Uncontrolled Asthma

**DOI:** 10.1155/2018/5698212

**Published:** 2018-11-05

**Authors:** Diego Bagnasco, Marco Caminati, Matteo Ferrando, Teresita Aloè, Elisa Testino, Giorgio Walter Canonica, Giovanni Passalacqua

**Affiliations:** ^1^Allergy & Respiratory Diseases, DIMI Department of Internal Medicine, University of Genoa, IRCCS AOU San Martino-IST, Genoa, Italy; ^2^Asthma Center and Allergy Unit, Verona University and General Hospital, Verona, Italy; ^3^Department of Internal Medicine, Respiratory Disease Clinic, IRCCS, Humanitas Clinical and Research Center, Humanitas University, Rozzano, Milan, Italy

## Abstract

The current developments of the new biological drugs targeting interleukin 5 (IL-5) and IL-5 receptor allowed to expand the treatment options for severe hypereosinophilic asthma. Clinicians will then be able to choose between antibodies targeting either circulating IL-5 or its receptor expressed on eosinophils and basophils. The available clinical trials consistently reported favorable results about the reduction of exacerbations rate, improvement in quality of life, and sparing of the systemic steroid use, with a favorable safety profile. Two of these new drugs are administered subcutaneously, mepolizumab every 4 weeks and benralizumab every 8 weeks, whereas reslizumab is given intravenously monthly on a weigh-based dose. In the future, the research actions will be involved in the identification of a single biomarker or multiple biomarkers for the optimal choice of biological agents to be properly prescribed.

## 1. Introduction

The recent change in the definition of asthma, from a unique disease characterized by a reversible airway obstruction to a heterogeneous disease (encompassing numerous phenotypes), prompted the research to look for more detailed pathogenic aspects, as endotypic targets, especially in uncontrolled or severe patients. In this regard, two main different phenotypes/endotypes of asthma could be distinguished, based on their inflammatory characteristics, that are, T helper lymphocyte type 2 (TH2)-high and TH2-low, depending on the predominance of TH2 cytokines [1]. The more and more detailed knowledge of the pathogenic mechanisms led to the discovery of “targeted” treatments to be used in subsets of non-controlled asthmatic patients. For historical and cultural reasons the best known pathogenic mechanism is mediated by eosinophils and IL-5. In fact, within the TH2-high asthma, allergic asthma (early onset, eosinophilic inflammation, and IgE mediated sensitization) remains a paradigm. Two main approaches were evaluated to block the action of IL-5 on eosinophil activation, survival, and migration. The first one is to block the circulating cytokine, and the second is to interfere with the IL-5 receptor alpha on eosinophils. Although the earliest experimental data on the effects of anti IL-5 in asthmatic patients were disappointing, with the only evidence that anti-IL-5 reduced eosinophils in peripheral blood, airways, and bone marrow, but no effects on airway hyperreactivity and bronchial allergen [2–5], a more accurate analysis of the data related to the first studies has allowed to highlight a better response to these drugs by those who had high levels of serum eosinophils. The use of these drugs has therefore been restricted to asthmatic patients with these biochemical characteristics.

The subsequent available clinical trials have shown a good efficacy in the above mentioned selected patients, with a favorable safety profile, for all of the three drugs [6].

## 2. IL-5 and Its Receptor Alpha

IL-5 is a 13-amino acid protein forming a 52-kDa homodimer, which has long been evaluated as a valuable therapeutic target [22], since it represents the main stimulus for growth, differentiation, survival, and activation of the cells [23]. IL-5, IL-3, and granulocyte-monocyte colony-stimulating factor (GM-CSF) all belong to the *β* common chain family and are able to bind a receptor involving the interleukin-5Ra and the common *β* subunit *β*c [24–26]. While IL-5 is more specifically involved in maturation and activation of eosinophils, IL-3 and GM-CSF have a more broad action, as survival factors for these cells [27]. Recently, IL-33 was found to play a non-negligible role in eosinophils homeostasis, through the activation of innate lymphoid cells type 2 (ILC2) [28].

## 3. IL-5 Antagonists

The awareness that IL-5 is involved in development, maturation, and action of eosinophils prompted the research to evaluate this cytokine as a possible therapeutic target in severe uncontrolled hypereosinophilic asthma. Two different mechanisms of action were identified, the former acting directly on IL-5 and the latter directly on IL-5 receptor alpha (IL-5Ra). Two different drugs are currently available to block IL-5: mepolizumab (recently commercialized with brand name Nucala; GSK) [2, 23] and reslizumab (proposed trade name Cinqair; Teva). Another biological drug blocking the IL-5 receptor alpha was approved by Food and Drug Administration (FDA) (Benralizumab, Fasenra) [29]. The antagonism to circulating IL-5 is intended to decrease the proliferation, maturation, and survival of eosinophils, whereas the ILR-a antagonism adds an antibody-dependent cell-mediated cytotoxicity (ADCC). Through this activity, essentially mediated by NK cells, Benralizumab can induce a peripheral and tissue destruction of both eosinophils and basophils [8]. The main end point, in clinical trials, regarding anti IL-5 or anti IL-5R drugs, ever was the reduction of exacerbations, the use of oral corticosteroids (OCS), and the effects on quality of life (QoL).

## 4. General Therapeutic Aspects

First clinical trials about these drugs evaluated the intravenous route of administration with the above mentioned results. After these trials a second route has been evaluated for all of these drugs, the subcutaneous. For mepolizumab and benralizumab, it was shown that both routes were equally effective, with a better safety profile and a more convenient use of the subcutaneous route. The same thing did not happen for reslizumab; indeed recently two phase III studies (evaluating subcutaneous reslizumab, 110 mg) did not meet the primary endpoint: the reduction of exacerbations in patients with severe uncontrolled hypereosinophilic asthma (blood eosinophils >300/mcL) in the first one (NCT02452190) and the reduction of daily systemic steroids in the second (NCT02501629) [30]. Therefore, so far, the optimal administration route for reslizumab remains the intravenous one that, on the other hand, allows to adjust the dose according body weight. Benralizumab is administered subcutaneously, like mepolizumab, at an 8-week time interval. The possibility to choose between two different routes (intravenous or subcutaneous) and a different times of administration (4 or 8 weeks) would allow the clinicians to more properly personalize the therapy according to the characteristics of the drugs and the patients' needs.

## 5. Exacerbations

The reduction in exacerbation rate and in the dose of systemic corticosteroids is usually the main endpoints in clinical trials, according to the definition of severe asthma [31]. Omalizumab (anti-IgE [32]) remained for 10 years the only biological treatment available for severe allergic asthma. The first regulatory trial with mepolizumab involved 61 subjects with a history of refractory hypereosinophilic asthma and frequent exacerbations. Patients received a monthly dose of 750 mg mepolizumab for one year. There was a reduction of the exacerbation rate in the active arm compared with the placebo group (2.0 vs. 3.4 mean exacerbations per subject; relative risk, 0.57; 95% confidence interval [CI], 0.32 to 0.92; P = 0.02) [33]. In another trial, the efficacy of mepolizumab in reducing exacerbations was tested in 20 adult patients with severe asthma. All patients received 750 mg mepolizumab or placebo for five months. At the end of the study, 12 exacerbations were recorded in the placebo group and two in the mepolizumab group (p=0.008) with a mean duration of exacerbation of 20 weeks in the active group and 12 weeks in the placebo one (P=0.003) [11]. The first trial with exacerbation rate formally defined as primary endpoint was DREAM. Six hundred and twenty-one patients with severe asthma and signs of eosinophilic inflammation were enrolled in this multicentric, double-blind, placebo-controlled trial. Three different intravenous dosages of mepolizumab (75 mg, 250 mg, and 750 mg) and placebo were administered. The exacerbations rate was significantly reduced in the active groups as compared to placebo (48% reduction; <0·0001) [7]. There was no difference in the efficacy and safety among the different doses in order to reduce exacerbations. SIRIUS study, where primary endpoint was the reduction of oral corticosteroids (OCS), evaluated also, as further endpoint, the exacerbations showing a significant reduction (32% less) in patients given mepolizumab compared with placebo [9]. The effects of mepolizumab, 75 mg intravenously or 100 mg subcutaneously, were assessed in the MENSA study. In this study, in the intravenous group the exacerbation rate was reduced by 32%, while in the subcutaneous group the decrease was 53% versus placebo [10].

For Reslizumab, the reduction of exacerbations was assessed in two duplicate, multicenter, double-blind, parallel-group, randomized, placebo-controlled (DBRPC) phase 3 trials. The drug (or placebo) was given at 3.0 mg/kg intravenously every 4 weeks for 1 year. The trial reported a significant reduction in asthma exacerbations in the active group (study 1: RISK ratio [RR] 0.50 [95% CI 0.37–0.67]; study 2: 0.41[0.28–0.59]; both p<0.0001). In addition, the time to first exacerbation was considerably longer in the active than in the placebo group [13].

Similarly to mepolizumab and reslizumab, several studies with Benralizumab evaluated the exacerbation rate reduction as primary endpoint. The results of a phase II DBRPC showed a reduction of exacerbations. A significant reduction of exacerbations rate (49%) and exacerbations requiring hospitalization (60%; 1.62 vs 0.65; P=.02), was also reported in another trial (3.59 vs 1.82; P=.01[18]). The SIROCCO study was a double-blind, parallel-group, placebo-controlled phase 3 clinical trial, where patients were assigned to 400 every four weeks and 398 every eight weeks Benralizumab 30 mg or placebo subcutaneously. The active drug reduced the asthma exacerbation rate, during the year of observation both in the 4-week (RISK ratio 0.55, 95% CI 0.42–0.71; p<0.0001) and in the 8-week group (0.49, 0.37–0.64; p<0.0001) [19]. Exacerbation reduction has been evaluated also in CALIMA study, with the same inclusion criteria and dosing regimens of SIROCCO, showing similar results with a significantly lower annual exacerbation rate both in the group treated with 30 mg every 4 weeks (0.60 [95% CI 0.48–0.74], rate ratio 0.64 [95% CI 0.49–0.85], p=0.0018, n=241), and in the one treated every 8 weeks, compared with placebo [20]. The latest published clinical trial on Benralizumab in severe hypereosinophilic patients (ZONDA) reported a significant reduction in exacerbation rate in both groups (30 mg/4 weeks or 30 mg/8 weeks), with a decrease of 55% in patients treated every 4 weeks, and 70% in those who assume therapy every 8 weeks, versus the one treated with placebo [21].

## 6. The OCS Sparing Effect

A special attention was recently devoted to steroid-dependent patients; this was due to the well-known burden of steroid related side effects (diabetes, hypertension, obesity, cataract, etc.) [34]. In one study, all enrolled patients received a mean daily dose of 10 mg of prednisone both in placebo and in mepolizumab group. After the treatment period, the active group had a mean reduction of their dose of 83.8 ± 33.4%, as compared to 47.7 ± 40.5% in the control group (P=0.04) [11]. In the SIRIUS study, in a cohort of 135 patients with severe eosinophilic asthma those receiving mepolizumab could reduce the dose of oral steroids 2.65 times versus those receiving placebo (95% CI, 1.25 to 4.56; P=0.008) [9]. A trial where reslizumab's OCS sparing effect has been indicated as primary endpoint is actually ongoing (NCT02501629). Preliminary results of this trial have been recently published in an official note, showing the failure of the drug in order to reduce daily OCS dose [30]. The effect of Benralizumab, on the reduction in the OCS dose, has been recently published. The study design involved 28 weeks of Benralizumab (30 mg subcutaneously, either every 4 weeks or every 8 weeks [with the first three doses administered every 4 weeks]) versus placebo. For both active groups, the median OCS reduction at week 28 was 75% in active patients compared with 25% in the placebo group. The percentage of patients that could completely withdraw their OCS daily dose (secondary endpoint) was 56% in the every 4 weeks and 52% in the every 8 weeks administration, as compared with 19% in the placebo group [21].

## 7. Quality of Life (QoL)

In addition to exacerbations, lung function, and safety, the effects on QoL are also relevant when a new drug is evaluated. Within the above mentioned trials with mepolizumab, Haldar et al. evaluated the effect of the medication on QoL, measured by the Asthma Quality of Life Questionnaire (AQLQ). After treatment, AQLQ improved from 0.55 in the active group to 0.19 [33]. On the other hand, the DREAM study failed to demonstrate a statistically significant effect on FEV_1_ and AQLQ [7]. MUSCA is the most recent large trial assessing health-related quality of life (HRQOL) in severe asthmatic patients as primary endpoint. It is a randomized, double blind, placebo-controlled, parallel group, multicenter, phase 3b trial, with 274 mepolizumab patients and 277 placebo patients enrolled. Inclusion criteria were a history of at least two exacerbations in the previous year treated with corticosteroids. The St George's Respiratory Questionnaire (SGRQ) was used to assess the changes in HRQOL. At week 24 a significant improvement in symptoms in the active group was documented as compared with placebo [12]. One of the first trials with reslizumab evaluated the effect of 0.03 mg/kg, 0.1 mg/kg, 0.3 mg/kg, or 1.0 mg/kg or placebo, in severe asthmatic patients with persistent symptoms, needing OCS and high-dose of inhaled steroids. With the 1 mg/kg dose a decrease of peripheral eosinophils was seen, but no improvement in symptoms [35]. A more recent study demonstrated a significant reduction in ACQ-7 in patients treated with reslizumab vs placebo (71% vs. 57%; p=0.01) [15]. In a similar trial, where reslizumab was given at 0.3 mg/kg and 3 mg/kg, an improvement in QoL, measured with ACQ, ACQ-5, ACQ-6, and AQLQ, was demonstrated with the highest dose [16]. Concerning Benralizumab, an improvement in QoL (ACQ-6 and AQLQ) was seen, especially in subjects with baseline blood eosinophils ≥300 cells per *μ*L [19, 20]. An improvement in QoL was confirmed also in the ZONDA study, where active arm ACQ-6 scores decreased by 0.55 points vs. placebo (p=0.001) [21] (Table 1).

## 8. Safety

The general safety of anti-IL biologicals, as assessed in controlled trials, has been described and reviewed elsewhere [36, 37]. Nonetheless, other special safety aspects have been proposed as a matter of discussion.

For instance, the defensive role of eosinophils, especially against helminthic infections, is well known, and for this reason the effects of the drug-induced depletion of eosinophils were debated. Indeed, several studies in guinea pigs treated with eosinophils antiserum failed to demonstrate an increased risk of helminth infestation [38]. Also, the long term (more than 6 months) treatments in mice and primates with antibodies abating eosinophils did not demonstrate any observable adverse effects [39, 40]. The most common non-serious AE in clinical trials with mepolizumab were injection site reaction, headache, nasopharyngitis, and upper respiratory tract infection, not different from placebo groups [7, 9–12, 33]. In the largest clinical trials, some serious adverse events (SAE) were described, mainly worsening of asthma [5, 9]. Three fatal events, all in the intravenous mepolizumab groups, were reported, but none of these cases were considered as drug-related [7]. No fatal event was reported with the subcutaneous route. With reslizumab, four cases of anaphylactic reaction were described in two different trials [13, 15]. Also for reslizumab the main SAEs were worsening of asthma, followed by pneumonia [10, 13, 14, 16]. One patient in the placebo group died due to multiple-drug overdose [13]. Worsening of asthma appeared as the most frequently described SAE also in the benralizumab studies [20, 21]. In those trials, some fatal events (due to pneumonia, acute cardiac failure, cerebral hemorrhage, asthma, opioid overdose, suicide, road traffic accident, acute myocardial infarction, colon neoplasm, and unknown causes) were in the active group patients. Pulmonary embolism, myocardial infarction, and unknown causes were in patients treated with placebo [17, 19–21].

## 9. Anti-IL-5 Treatments: Practical Aspects and Problems

All the available (or soon available) IL-5 antagonists (mepolizumab, reslizumab, and benralizumab) show a favorable cost-to-benefit profile, in addition to clinical efficacy and biological effects. When all drugs will be marketed, we could choose between different kinds of administration (intravenous or subcutaneous) and administration frequency (4 or 8 weeks). For administration route, it has been already said that reslizumab, at least at the moment, remains with the only intravenous way; regarding the frequency of administration it is interesting that benralizumab could be dispensed every 8 weeks. In published clinical trials and documents no pathophysiological motivation was provided to explain the possibility to doubling administration time; however it is evident that, with the same efficacy results shown in the CALIMA and in the SIROCCO study [19, 20] at 4-week and 8-week administration, the pathway every 8 weeks is economically and operationally more sustainable for this drug.

Regardless of the route and frequency of administration, in the main clinical trials all the above mentioned IL-5 antagonists have proven to be more effective in the severe form of asthma with high levels of blood eosinophils (300 cells/mm^3^ for Benralizumab and mepolizumab and 400 cells/mm^3^ for reslizumab). The fact that it has been proven that all three drugs are more effective in the same types of subjects (severe asthmatics with serum hypereosinophilia), once on the market, could be a problem. Indeed if we have three drugs with similar patient targets, and a very similar efficacy between the different molecules, it will be difficult to choose [41]. Moreover, regarding efficacy, it has been shown that all anti-IL-5 drugs not only need high blood eosinophils levels, but also highlight the fact that the number of eosinophils present in patients' serum correlates with the effect of the drug administered. Indeed a secondary analysis of MENSA and DREAM studies demonstrates that the reduction in exacerbations rate is positively associated with increasing blood eosinophil count at baseline [42].

Nevertheless, the clinical aspects (symptoms, pulmonary function, exacerbations, and exhaled nitric oxide) still perceived the only (and insufficient) predictive biomarkers to guide the prescription of such expensive drugs. The coexistence of chronic rhino sinusitis with nasal polyposis could be a criterion for the choice of one drug or other biological drugs. Also, the route of administration (intravenous or subcutaneous) and the possibility of adjusting the dosage would be possible suggestions for clinicians. In addition to IL-5 antagonists, other biological drugs such as anti IL-4 and IL-13 [43] were proposed, although the recent preliminary results on Tralokinumab (anti IL-13) displayed unfavorable results (STRATOS 2 (exacerbations) and TROPOS reduction in OCS use) in severe asthma [44]. The possible answer is biomarkers, some biological or clinical samples, able to drive clinician to the choice [45]. Notwithstanding some studies proposed several biomarkers, such as serum total IgE levels (IgEs) [46], FeNO, blood, and sputum eosinophil count [47, 48]; there is not a certain role of these samples as predictive indicator of response for one or the other drug. Other biological samples have been evaluated, like periostin, both in bronchoscopy biopsies [49] and in less invasive way [50], and are still under evaluation. Given that clinical trials have shown promising efficacy for all three drugs described, as already stated, once all these drugs are marketed the challenge could be which one to choose to provide an increasingly personalized medicine and choose the one that preventively could be the best. At the moment, due to the fact that no certain biomarker has been discovered, to choose the better drug for our patients, we could use a more clinic approach and we can rely on what emerged from trials and literature. Several authors suggest that the single dosage of mepolizumab could be a limit in overweight-obese patients, and the possibility to a weight-adjustment could be useful. About that Mukherjee and coauthors have described a trial where ten patients, demonstrating a non-fully response to mepolizumab after 1 year of administration, after a 1-year period of wash-out, have been treated with 3.0 mh/Kg of reslizumab with an increase of QoL and decrease of sputum and blood eosinophilia after 4 months of administration [51]. This could be used as a discriminant to choose one drug rather than another. On the other hand mepolizumab seems to have the same efficacy in the patients treated in the trials both at the marketed dosage (100 mg) and at higher doses, confirming its effectiveness regardless of weight, making it safe and effective to be prescribed independently of the body mass index (BMI) value [7, 10]. Regarding the anti-receptor drug, benralizumab, an advantage could be the periodicity of administration; indeed, after a “run-in” period where for three months the dosage is at 4-week frequency, the drug will be injected every 8 weeks. This therapeutic scheme could be advantageous due to the fact that the intake of the drug with a 8-week frequency would decrease the indirect costs (lost work days, visits made, etc.), and, depending on the cost of the drug agreed upon with the local health ministries, also the direct ones. We could have greater clarity on the choice of drugs with the development of single biomarkers or panels of laboratory and clinical parameters, and real life studies.

## 10. Conclusions

The wide variety of anti-IL-5 antagonists or IL-5 receptor blockers allow to have alternative treatment options for patients with severe hypereosinophilic patients. All the three drugs herein reviewed displayed a good safety profile, and a favorable clinical efficacy in the selected patients. It remains true that we do not still have reliable predictive markers to detect which single patient will respond individually to each of such expensive treatments. Also, the different routes of administrations would provide clinicians with the opportunity to choose the drug according to drug characteristic and patient's needs. At present, the best biomarker in patient eligible for anti-IL-5 or IL-5ra is blood eosinophils, exhaled nitric oxide, and clinical phenotyping (age of onset of asthma, atopy, and presence of nasal polyposis). Predictive biomarkers allowing a better prescription of a personalized medicine are needed, although the introduction in clinical practice of novel biologics targeted to severe asthma represents a step forward.

## Figures and Tables

**Table 1:** Exacerbations, OCS sparing, QoL, and safety of main clinical trials about anti-IL-5 and anti-IL-5Ra.

		EXACERBATION	OCS sparing	QoL	SAFETY
MEPOLIZUMAB	Pavord et al. [7]	exacerbation (48%with 75mg dose/39% with 250mg dose/52% with 750mg dose)	not performed	no improvement of QoL (tests using AQLQ )	common: headache, nasopharyngitis, infusion related reaction Serious: 3 death (1 septic shock after acute pancreatitis, fatal asthma attack, suicide)
	FloodPage et al. [8]	no significant difference between groups (16% placebo, 18 % 250 mg, 10% 750 mg)	not performed	improvement in treated patients, with all dose	serious: placebo (bladder carcinoma, unintended pregnancy, and asthma exacerbation); 250 mg of mepolizumab (hydrocephalus/cerebrovascular disorder, constipation, and gastrointestinal disturbance); 750 mg of mepolizumab (asthma exacerbation)
	Bet et al. [9]	32% exacerbation less	reducing daily dosage ( 2,65 times more than patient receiving placebo)	small change in ACQ	common: headache, nasopharyngitis, infusion related reaction Serious: asthma exacerbation, pneumonia (both in placebo group)
	Ortega et al. [10]	exacerbation (with intravenous medication, 47%; with subcutaneous administration, 53%)	not performed	improvement in QoL	common: headache, nasopharyngitis, upper respiratory tract infection
	Nair et al. [11]	reduction of exacerbations in treated patients	reducing daily dosage	not performed	common: 1 patient with shortness of breath, 1 with aches and tiredness serious: 1 death in placebo group
	Chupp et al. [12]	reduction of exacerbations of 58%	not performed	improvement of QoL (tests using SGRQ)	common: headache, nasopharyngitis, urticaria, arthralgia, arrhythmias, injection-site reaction Serious: 8 arrhythmias (2 in mepolizumab and 6 in placebo), 1 deep venous thrombosis in mepolizumab group

RESLIZUMAB	Castro et al. [13]	exacerbation (people without exacerbation: 44%with placebo, 61% with reslizumab)	no improvement in OCS sparing	improvement of QoL (test using ACQ)	common: nasopharyngitis, upper respiratory tract infection serious: 2 anaphylactic reaction
	Castro et al. [14]	exacerbation (people without exacerbation:52% with placebo,73% with reslizumab)	no improvement in OCS sparing	improvement of QoL (test using AQLQ and ACQ-7)	common: nasopharyngitis serious: pneumonia, worsening of asthma
	Corren et al. [15]	not performed due to the short observation period (16 weeks)	not performed	improvement of QoL (test using ACQ-7)	serious: 2 anaphylactic reactions, 1 colon cancer (all in reslizumab group)
	Bjemer et al. [16]	not performed due to the short observation period (16 weeks)	not performed	improvement of QoL (test using ACQ and AQLQ)	serious*: placebo (*1 acute myocardial infarction), 3 asthma exacerbations, 1 sinusitis, 1 pneumonia, 1 road traffic accident and 1 rib fracture

BENRALIZUMAB	Castro et al. [17]	no difference in noneosinophilic patients between benralizumab and placebo. Reduction in eosinophilic patients.	not performed	improvement in AQLQ in people with at least 300 eosinophils/mmc	serious: 100 mg dosage acute cholecystitis, herpes zoster, polyarteritis nodosa, and uterine leiomyoma 20 mg dosage: erythema nodosum
	Nowak et al. [18]	exacerbation (49%) and exacerbation requiring hospitalization (60%)	not performed	no significant improve in ACQ and AQLQ	common: headache, asthma, dizziness, cough, pyrexia, bronchitis, anxiety, muscle spasm serious*:* tachycardia and anxiety
	Bleecker et al. [19]	exacerbation in Q4W and Q8W	not performed	improvement in patients with baseline	common*:* nasopharyngitis, worsening of asthma serious: allergic granulomatous angiitis, panic attack, paraesthesia
	Fitzgerald et al. [20]	exacerbation in Q4W and Q8W	not performed	blood eosinophils ≥300 cells per *μ*L	common: nasopharyngitis, worsening of asthma serious: urticaria, asthma, herpes zoster, chest pain
	Nair et al. [21]	exacerbation (55% with 30 mg dose every 4 weeks; 70% with 30 mg dose every 8 weeks)	interruption of OCS (56% of who received drug every 4 weeks and 52% of 8 weeks administration, as compared with 19% treated with placebo)	improvement in patients with baseline	serious: worsening of asthma, pneumonia, hearth failure, pericarditis (placebo). Two case of death in Q8W due to pneumonia and acute cardiac failure.
